# Improving outcomes for asthma patients with allergic rhinitis: conclusions from the MetaForum conferences

**DOI:** 10.1186/1471-2466-6-S1-S7

**Published:** 2006-11-30

**Authors:** David Price, Stephen T Holgate

**Affiliations:** 1 Department of General Practice and Primary Care, University of Aberdeen, Foresterhill Health Centre, Westburn Road, Aberdeen AB25 2AY, UK; 2 Infection, Inflammation and Repair AIR Division, Level F, South Block, MP810, Southampton General Hospital, Tremona Road, Southampton SO16 6YD, UK

## 

The two MetaForum conferences provided an opportunity for presentation of current information on asthma and allergic rhinitis, as well as a forum for discussion by European experts of important issues in the field of allergy and asthma [1]. Several key points were the focus of the talks and discussions and led to the development of consensus recommendations at the end of each conference. These topics remain the focus of active investigation as well as being very relevant today.

Results of epidemiologic studies indicate that allergic rhinitis is a risk factor for asthma, that allergic rhinitis and asthma often occur in association, and that having comorbid allergic rhinitis is a marker for the presence of more difficult to control asthma and greater use of resources for asthma [2]. Patients enrolled in the international survey reported in the present supplement often experienced a worsening of asthma symptoms when allergic rhinitis symptoms worsened [3]. Moreover, inflammatory processes are very similar in asthma and allergic rhinitis [4].

These findings support the Allergic Rhinitis Impact on Asthma recommendation that all patients with asthma be checked for allergic rhinitis, and vice versa [5]. Treatment plans need to take into account the presence of both conditions. More generally, there is a need to promote the use of combined therapies that are safe and effective for treating symptoms of both asthma and allergic rhinitis and that address the inflammatory nature of these two conditions affecting the 'one airway.' Active study continues to better characterize the burden of comorbid asthma and allergic rhinitis [6-8], as well as the potential benefits of concomitantly treating both conditions [9].

The structured review of patient surveys reported in the present supplement [10] suggests that asthma remains poorly controlled for many adults and children. Patients tolerate symptoms and have low expectations of asthma treatment; communication between patients and their physicians could be improved; and physicians often inaccurately assess disease severity and asthma control. Results of a recent review of pediatric studies worldwide support these findings and suggest that asthma guidelines are often not followed [11].

Better education of physicians about asthma and allergic rhinitis and better communication between physicians and patients are essential to improving asthma control. The final paper of the present supplement examines recent asthma guidelines and proposes possible ways in which they could be improved [12]. Physicians need simple and practical tools to facilitate the diagnosis and assessment of asthma in addition to identifying the factors responsible for poor control such as associated allergic rhinitis, limited adherence, and smoking behavior. A simple questionnaire to assess potential asthma symptoms in patients with allergic rhinitis has recently been described [13]. Rapid dissemination of information about such tools is needed once they have been developed and validated.

Finally, the development of country-specific guidelines or, ideally, local guidelines for each region would provide more practical solutions for asthma care and would account for factors, such as social factors and costs, that influence patient choice and adherence to therapy. These issues require coordinated efforts between specialists in the field and primary care physicians, as most medical care for asthma and allergic rhinitis is delivered in a primary care setting.

## Competing interests

STH has received fees for lectures from Novartis and Merck Sharp & Dohme and is a consultant for Novartis, MRL, Almiral Prodesfarma, Rotta Pharm., Cambridge Antibody Technology, Amgen, Wyeth, UCB/Celltech, Avontec and Synairgen. DP has received honoraria for speaking at sponsored meetings from the following companies marketing respiratory products: 3M, Altana, AstraZeneca, Boehringer Ingelheim, GlaxoSmithKline, IVAX, Merck Sharp & Dohme, Novartis, Pfizer and Schering-Plough. DP has also received honoraria for advisory panels with 3M, Altana, AstraZeneca, Boehringer Ingelheim, GlaxoSmithKline, IVAX, MSD, Novartis, Pfizer and Schering-Plough. DP or his research team have received funding for research projects from 3M, Altana, AstraZeneca, Boehringer Ingelheim, GlaxoSmithKline, IVAX, Merck, Sharp & Dohme, Novartis, Pfizer, Schering-Plough, Viatris.
